# Supplementary material for: Comparison of domain adaptation techniques for white matter hyperintensity segmentation in brain MR images
Source: Med Image Anal. 2021 Dec;74:102215. doi: 10.1016/j.media.2021.102215 (PMC8573594; doi:10.1016/j.media.2021.102215)
Supplement: Supplementary Data S1 — Supplementary Raw Research Data. This is open data under the CC BY license http://creativecommons.org/licenses/by/4.0/ [file mmc1.pdf]

Supplementary material

1 Determination of source and target datasets

The five datasets used in this work were acquired using different scanners and sequences and therefore have different intensity characteristics and resolutions: MICCAI WMH segmentation challenge 2017 training dataset (VU Amsterdam, NUHS Singapore and UMC Utrecht cohorts), NDGEN and OXVASC (see main text for details). For performing our domain adaptation (DA) experiments, we classified the available datasets into two domains. Rather than considering each dataset as an individual domain, we considered only two domains (source and target) for our experiments. This is because the datasets have varying degrees of similarity among them and also because, given the limited amount of data for each dataset (for training and testing), treating them as individual domains would be difficult and give unreliable results. For deciding the source and target datasets, we determined the homogeneity of image-level characteristics among the above 5 datasets, using a domain discriminator network.

Data preparation: We used FLAIR and T1 images, and applied the same preprocessing pipeline as that of the main DA experiments on the datasets - reorientation to the MNI space, brain extraction using FSL BET, bias-field correction using FSL-FAST, cropping the FOV tighter to the brain and Gaussian normalisation of intensity values. We extracted 2D axial slices for training and testing (to be consistent with the 2D networks used in the DA experiments) cropped to the dimension of 128 $\times$ 192 voxels.

Network architecture: As shown in figure S1, our domain discriminator network consists of an encoder connected to fully-connected layers and a final softmax layer, which outputs the probability values of predicted domains. Our network closely follows the VGG architecture [1], with 2 convolutional layers instead of 3 at each level. The network consists of 8 convolutional layers using 3 $\times$ 3 filters, with a 2 $\times$ 2 max-pooling layer after every 2 convolutional layers. The final two fully-connected layers consist of 4096 and 1000 nodes, followed by a softmax layer. We used multi-class cross-entropy loss function for training our network.

Implementation details: We used the Adam optimiser with $\epsilon = 1 \times 10^{-4}$. We empirically used a batch size of 16 and learning rate of 1×10^{-3} and reduced it by a factor 1×10^{-1} every 3 epochs, until it reached 1×10^{-5}. Data augmentation was applied only using translation (x/y-offset $\in$ [-10, 10]) and rotation ($\theta \in$ [-10, 10]) to inflate the amount of data by the factor of 4. We avoided random noise injection and Gaussian filtering since that would distort the intensity and image-level characteristics of the slices, which is crucial for determining their domains. The hyperparameter values for the data augmentation transformations were randomly sampled from the closed intervals specified above using a uniform distribution. The network was trained on an NVIDIA Tesla V100 for 50 epochs, taking 25 seconds per epoch respectively. We used a criterion based on a patience value (number of epochs to wait for progress on validation set) of 20 epochs to determine model convergence (early stopping).

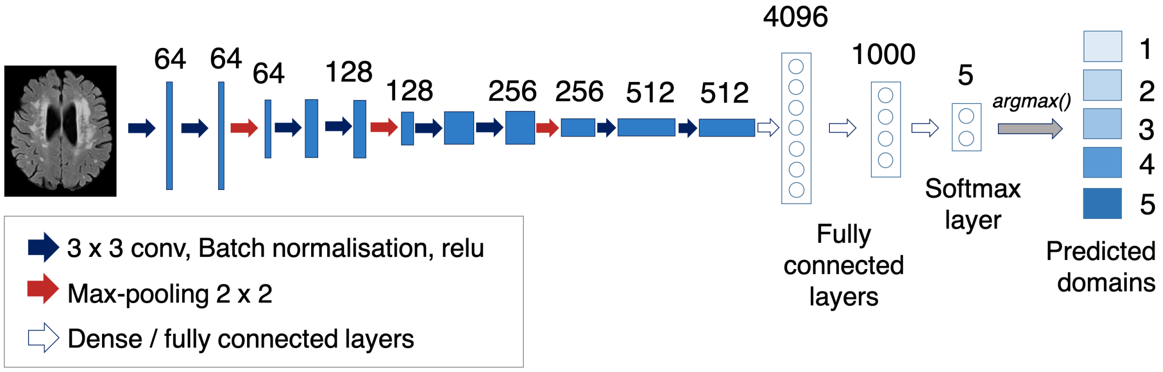

Figure S1: Domain discriminator network architecture.

Experiments and results: We combined all 5 datasets together with 5 labels: Amsterdam, Singapore, Utrecht, NDGEN and OXVASC, and performed 5-fold cross-validation on the combined dataset with training/validation/test split of 70/10/20% respectively, with the corresponding number of slices being $\approx$ 6200 (before augmentation)/900/1800 slices.

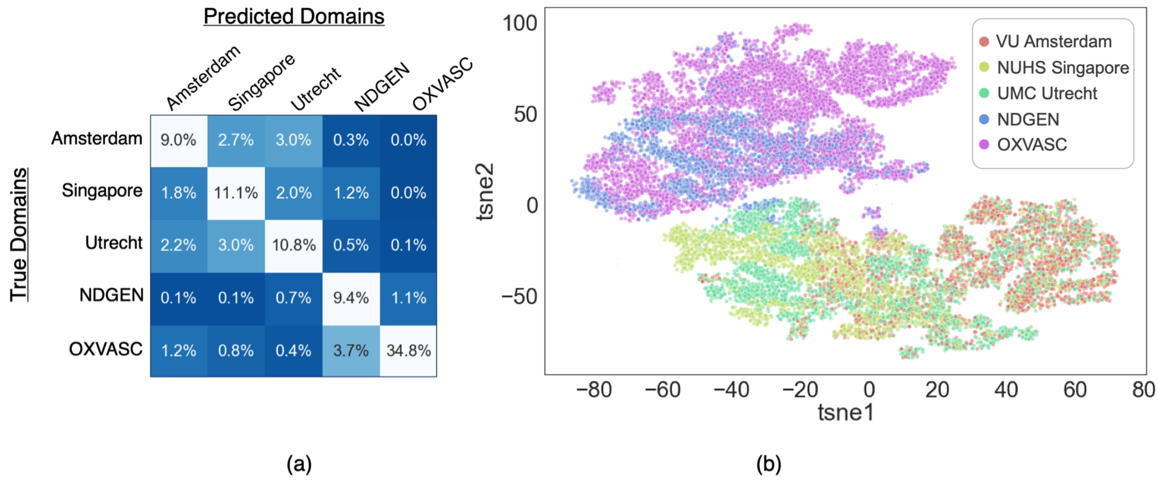

Figure S2: Results of the domain prediction. (a) Confusion matrix of predicted domain labels against the true domain labels (values indicated as the % of the total data). (b) t-SNE plot, based on results generated from FLAIR and T1 intensities, of the 5 datasets considered for DA experiments. Note that the source (MWSC cohorts) and the target (NDGEN and OXVASC) datasets' intensity characteristics are clearly separable.

Figure S2a shows the the confusion matrix of predicted domain labels against the true domain labels as percentages of the test data. From the figure, it can be seen that the misclassifications mainly occur among the MWSC cohorts and between the NDGEN and OXVASC datasets. The percentage of misclassified samples between MWSC and NDGEN/OXVASC datasets is smaller (4.9%) when compared to the misclassifications within MWSC cohorts (15.2%) and between the NDGEN and OXVASC datasets (6.5%). This indicates that MWSC cohorts are more similar to each other in their characteristics when compared to the NDGEN and OXVASC datasets. In particular, the misclassification of MWSC samples as OXVASC samples is quite low since the OXVASC dataset has different acquisition characteristics, with maximum spatial resolution in a different plane (coronal instead of axial) with respect to the other datasets. Hence, we combine the MWSC cohorts as the source domain datasets and

consider the combination of NDGEN and OXVASC as the target domain dataset. Figure S2b shows that the T-distributed Stochastic Neighbour Embedding (t-SNE) [2] plot of the 5 individual datasets, based on the FLAIR and T1 intensity values after preprocessing. As seen from the figure, while some overlap can be observed among the MWSC datasets and between NDGEN and OXVASC, the MWSC (source) and NDGEN/OXVASC (target) intensity characteristics are quite separable.

2 Sample results of various test strategies on a low lesion load subject

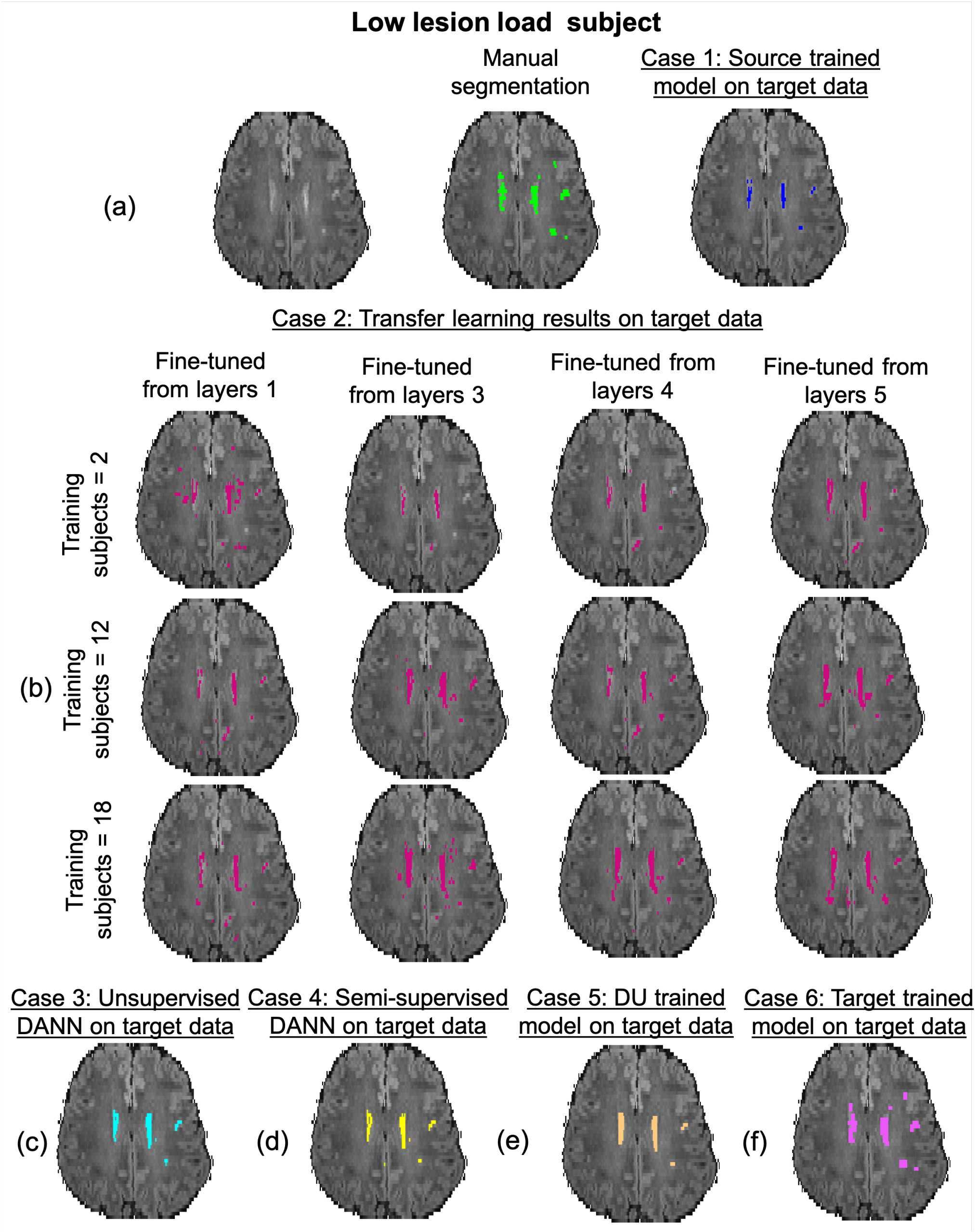

Figure S3: Sample results of domain adaption experiment test strategies on a low lesion load subject from the OXVASC (target) dataset, along with manual segmentation (green).

3 Performance evaluation metrics for DA strategies and permutation analysis of of linear models (PALM)

Table S1 shows the performance metrics for the comparison of various DA strategies, with respect to the target-trained case. Using permutation analysis of linear models (PALM), we performed two forms of multiple comparisons - (1) corrected across strategies and (2) corrected across both strategies and metrics. Table S2 shows p-values obtained after correcting for multiple comparisons across strategies (correction 1). We have also indicated the values (*) that were still significant after correction across both strategies and metrics (correction 2).

Table S1: Comparison of performance metrics for 5 DA strategies against the target-trained model on the target test dataset (from NDGEN and OXVASC) (median and interquartile range (IQR) values provided; the best median value(s) for each performance metric is highlighted in bold). For TL (strategy 2), we used the setting of 3 layers, 18 subjects for fine-tuning.

Perf. metrics	Strategy 1	Strategy 2	Strategy 3	Strategy 4	Strategy 5	Target-trained
SI value ↑	0.80 (0.68 - 0.86)	0.81 (0.73 - 0.87)	0.78 (0.66 - 0.92)	**0.84 (0.76 - 0.92)**	0.77 (0.70 - 0.83)	0.88 (0.84 - 0.98)
lAVD ↓	0.23 (0.10 - 0.39)	0.19 (0.15 - 0.26)	0.13 (0.06 - 0.24)	**0.10 (0.03 - 0.13)**	0.13 (0.05 - 0.18)	0.10 (0.06 - 0.17)
Cluster-wise F1-measure ↑	0.56 (0.43 - 0.63)	0.60 (0.52 - 0.72)	0.65 (0.55 - 0.81)	**0.79 (0.71 - 0.83)**	0.69 (0.58 - 0.75)	0.82 (0.58 - 0.98)
Cluster-wise TPR ↑	0.71 (0.59 - 0.85)	0.81 (0.72 - 0.87)	**0.86 (0.71 - 0.98)**	0.81 (0.77 - 0.90)	0.74 (0.64 - 0.83)	0.89 (0.66 - 0.99)
Voxel-wise TPR ↑	0.77 (0.70 - 0.89)	0.84 (0.75 - 0.91)	**0.87 (0.82 - 0.94)**	**0.87 (0.78 - 0.91)**	0.85 (0.67 - 0.98)	0.97 (0.82 - 0.98)
Voxel-wise FPR ↓	1.8 (1.3 - 5.2) $\times 10^{-4}$	1.8 (0.9 - 3.5) $\times 10^{-4}$	**0.8 (0.3 - 1.6) $\times 10^{-4}$**	1.3 (0.5 - 3.3) $\times 10^{-4}$	1.7 (1.0 - 2.8) $\times 10^{-4}$	1.2 (0.6 - 4.7) $\times 10^{-4}$

4 Swapping the source and target domains and evaluating the performance of DANN and semi-DANN models

We originally considered the MWSC dataset as the source dataset, and the NDGEN + OXVASC datasets as the target domain dataset (as explained in the main manuscript). Our aim in this experiment is to determine the effect of the size and image characteristics of individual domains on the best performing adversarial adaptation techniques, DANN and semi-DANN. Towards this aim, we swapped the source and target domain datasets, trained the DANN and semi-DANN models on the swapped datasets and evaluated as specified in the main manuscript (sections 2.4.3 and 2.4.4). Table S3 shows the performance metrics of the DANN and semi-DANN models on the MWSC and NDGEN + OXVASC test subjects for both original and swapped cases.

From the table, we observed that while DANN model is susceptible to slight changes in the performance (however, none of them significant except voxel-wise FPR), semi-DANN provided a consistent performance after domain swapping, without any significant difference in performance. This could be due to the fact that semi-DANN learns the characteristics of both domains, and hence less affected by the swapping of source and target domain datasets.

5 Additional strategy: Train on the source and target domain training datasets combined together

We train the baseline model (TrUE-Net) on the source and target datasets individually in strategy 1 and target trained case respectively. However, it would be good to determine the

Table S2: Results of t-test results between individual combinations of test strategies on the target dataset corrected for multiple comparisons across strategies (correction 1) using Permutation Analysis of Linear Models (PALM [3]) (* indicates values that were still significant after correction across both strategies and metrics (correction 2)), p < 0.05 highlighted in bold. Cl. F1 - Cluster-wise F1 measure, Cl. TPR - Cluster-wise true positive rate (TPR), Vox. TPR - Voxel-wise TPR, Vox. FPR - Voxel-wise false positive rate.

	**Strategy 2**	**Strategy 3**	**Strategy 4**	**Strategy 5**	**Target-trained**
**Strategy 1**	SI: p = 0.58<br>lAVD: p = 0.80<br>Cl. F1: p = 0.65<br>Cl. TPR: p = 0.23<br>Vox. TPR: **p = 0.04**<br>Vox. FPR: p = 0.84	SI: p = 0.64<br>lAVD: p = 0.15<br>Cl. F1: p = 0.19<br>Cl. TPR: **p = 0.03**<br>Vox. TPR: **p = 0.03**<br>Vox. FPR: **p = 0.001**	*SI: **p = 0.01**<br>lAVD: p = 0.29<br>*Cl. F1: **p <0.001**<br>Cl. TPR: **p = 0.012**<br>Vox. TPR: p = 0.09<br>Vox. FPR: p = 1.00	SI: p = 0.72<br>lAVD: **p = 0.04**<br>Cl. F1: **p = 0.03**<br>Cl. TPR: p = 0.36<br>Vox. TPR: p = 0.73<br>Vox. FPR: p = 0.50	SI: **p = 0.02**<br>lAVD: **p = 0.02**<br>*Cl. F1: **p = 0.009**<br>*Cl. TPR: **p <0.001**<br>*Vox. TPR: **p = 0.002**<br>Vox. FPR: p = 0.43
**Strategy 2**		SI: p = 1.00<br>lAVD: p = 0.29<br>Cl. F1: p = 0.92<br>Cl. TPR: p = 1.00<br>Vox. TPR: p = 0.46<br>Vox. FPR: p = 0.33	SI: p = 0.81<br>lAVD: p = 0.52<br>*Cl. F1: **p = 0.02**<br>Cl. TPR: p = 0.99<br>Vox. TPR: p = 0.26<br>Vox. FPR: p = 0.99	SI: p = 0.99<br>lAVD: p = 0.76<br>Cl. F1: p = 0.09<br>Cl. TPR: p = 0.62<br>Vox. TPR: p = 0.25<br>Vox. FPR: p = 0.99	SI: p = 0.86<br>lAVD: p = 0.61<br>Cl. F1: p = 0.15<br>Cl. TPR: p = 1.00<br>Vox. TPR: p = 0.99<br>Vox. FPR: p = 1.00
**Strategy 3**			SI: p = 0.85<br>lAVD: p = 0.98<br>Cl. F1: p = 0.25<br>Cl. TPR: p = 1.00<br>Vox. TPR: p = 0.92<br>Vox. FPR: p = 0.18	SI: p = 0.99<br>lAVD: p = 1.00<br>Cl. F1: p = 0.95<br>Cl. TPR: p = 0.51<br>Vox. TPR: p = 0.90<br>Vox. FPR: **p = 0.004**	SI: p = 0.77<br>lAVD: p = 1.00<br>Cl. F1: p = 0.17<br>Cl. TPR: p = 1.00<br>Vox. TPR: p = 0.38<br>Vox. FPR: p = 0.24
**Strategy 4**				SI: **p = 0.97**<br>lAVD: p = 0.98<br>Cl. F1: p = 0.86<br>Cl. TPR: p = 0.19<br>Vox. TPR: p = 0.59<br>Vox. FPR: p = 0.86	SI: p = 0.99<br>lAVD: p = 0.99<br>Cl. F1: p = 0.99<br>Cl. TPR: p = 1.00<br>Vox. TPR: p = 0.92<br>Vox. FPR: p = 0.99
**Strategy 5**					SI: p = 0.96<br>lAVD: p = 0.99<br>Cl. F1: p = 0.94<br>Cl. TPR: p = 0.39<br>Vox. TPR: p = 0.37<br>Vox. FPR: p = 0.99

Strategy 1: source-trained model applied on the target test dataset; Strategy 2: transfer learning (3 layers, 18 subjects); Strategy 3 : domain adversarial training of neural networks (DANN); Strategy 4: Semi-supervised DANN; Strategy 5: domain unlearning; Target-trained: target-trained model applied on the target test dataset.

inherent domain adaptation of TrUE-Net by training the model on the source and target

Table S3: Results of DANN and semi-DANN strategies when the source and target datasets are swapped. The corresponding p-values for the differences between the two cases, determined using Wilcoxon signed rank test results are shown (p < 0.05 highlighted in bold). Cl. F1 - Cluster-wise F1 measure, Cl. TPR - Cluster-wise true positive rate (TPR), Vox. TPR - Voxel-wise TPR, Vox. FPR - Voxel-wise false positive rate.

		ORIGINAL: Source: MWSC, Target: NDGEN + OXVASC		SWAPPED: Source: NDGEN + OXVASC, Target: MWSC		P-value (between ORIGINAL and SWAPPED cases)	
	**Perf. metrics**	**MWSC test data**	**NDGEN + OXVASC test data**	**MWSC test data**	**NDGEN + OXVASC test data**	**MWSC test data**	**NDGEN + OXVASC test data**
Strategy 3: DANN	**SI value**	0.91 (0.73 - 0.94)	0.78 (0.66 - 0.92)	0.79 (0.75 - 0.92)	0.82 (0.70 - 0.89)	0.30	0.46
	**AVD (%)**	11.2 (3.9 - 23.3)	17.1 (7.7 - 35.2)	15.8 (5.4 - 30.1)	12.6 (6.6 - 36.2)	0.96	0.74
	**Cl. F1**	0.82 (0.61 - 0.93)	0.65 (0.55 - 0.81)	0.74 (0.68 - 0.82)	0.69 (0.58 - 0.87)	0.68	0.64
	**Cl. TPR**	0.90 (0.75 - 0.93)	0.86 (0.71 - 0.99)	0.83 (0.71 - 0.94)	0.90 (0.76 - 0.99)	0.23	0.55
	**Vox. TPR**	0.89 (0.83 - 0.96)	0.87 (0.82 - 0.94)	0.86 (0.82 - 0.94)	0.88 (0.82 - 0.95)	0.25	0.56
	**Vox. FPR**	8.9 (3.8 - 14.7) $\times 10^{-5}$	8.2 (3.8 - 16.0) $\times 10^{-5}$	17.2(4.3 - 36.0)$\times 10^{-5}$	7.1 (2.7 - 13.5) $\times 10^{-5}$	**0.001**	0.46
Strategy 4: Semi-DANN	**SI value**	0.86 (0.79 - 0.92)	0.84 (0.76 - 0.92)	0.81 (0.77 - 0.94)	0.82 (0.76 - 0.94)	0.84	1.00
	**AVD (%)**	8.5 (3.0 - 15.8)	12.8 (6.2 - 22.1)	12.8 (2.7 - 23.9)	6.2 (3.2 - 17.3)	0.71	0.12
	**Cl. F1**	0.81 (0.63 - 0.87)	0.79 (0.71 - 0.83)	0.76 (0.69 - 0.85)	0.80 (0.76 - 0.82)	0.76	0.62
	**Cl. TPR**	0.87 (0.79 - 0.91)	0.81 (0.77 - 0.90)	0.90 (0.71 - 0.92)	0.82 (0.78 - 0.88)	0.88	1.00
	**Vox. TPR**	0.87 (0.83 - 0.90)	0.87 (0.78 - 0.91)	0.87 (0.82 - 0.91)	0.87 (0.82 - 0.90)	0.99	0.63
	**Vox. FPR**	17.8(11.2 - 39)$\times 10^{-5}$	13.3 (5.1 - 33.7)$\times 10^{-5}$	16.0 (7.2 - 43.8)$\times 10^{-5}$	13.3 (7.5 - 42.7)$\times 10^{-5}$	0.68	0.62

training datasets combined together. Also, this strategy would also provide a good benchmark to compare transfer learning (TL) since the TL also involves both the domains. In this strategy, we trained the TrUE-NET model on the combination of source and target domain training datasets (training parameters in sec. II-E) and tested the model directly on the target domain test datasets using performance metrics specified in sec. II-G.

5.1 Results

We achieved median SI = 0.82, lAVD = 0.19, cluster-wise F1-measure = 0.61, cluster-wise TPR = 0.79, voxel-wise TPR = 0.80 and voxel-wise FPR = 1.9×10^{-4} on the target test dataset. These results were better than those of source-trained model (strategy 1) with the difference significant for SI value (p = 0.03), lAVD (p = 0.04), voxel-wise FPR (p = 0.03) using Wilcoxon signed rank test. On comparing the results with TL (strategy 2), we observed that TL provided better results when compared to this strategy. Wilcoxon signed rank test results between this strategy and TL provides the following p-values: SI = 0.78, AVD < 0.001, cluster-wise F1-measure < 0.001, cluster-wise TPR = 0.65, voxel-wise TPR = 0.06 and voxel-wise FPR = 0.47, with the differences in AVD and cluster-wise F1-measure significantly different. This is because in the TL strategy, the model weights were fine-tuned using target training data to provide better WMH segmentation on the target test data. On the other hand, the model trained on combined data needed to learn the features that would provide the optimal performance in both domains (with the trade-off in the target domain performance). Therefore the performance of this strategy tends to get worse on the both domains with the increase in the domain shift (i.e. differences in data characteristics between domains).

6 Comparing the source-trained model performance with the TL case with a few subjects from the target domain

As a part of the TL analysis, we did perform fine-tuning using different number of training subjects from target domain starting from 2 to 18, incrementing in the steps of 2. On using 2-4 subjects from the target domain for training, the performance metrics for this strategy was better than the source-trained model (as reported in Table S4), but still lower than other DA methods where the model sees the entire labelled or unlabelled target data during training. However, as we increased the number of subjects for fine-tuning the performance increased progressively.

Table S4: Comparison of performance metrics for source-trained model with TL strategy, using fewer target subjects for fine-tuning, on the target test dataset (median and IQR values provided).

**Perf. metrics**	**Source-trained**	**TL (4-layers-2-subjects)**	**TL (4-layers-4-subjects)**
SI value ↑	0.80 (0.68 - 0.86)	0.81 (0.70 - 0.84)	0.82 (0.72 - 0.85)
lAVD ↓	0.23 (0.10 - 0.39)	0.20 (0.13 - 0.30)	0.20 (0.12 - 0.29)
Cluster-wise F1-measure ↑	0.56 (0.43 - 0.63)	0.61 (0.53 - 0.68)	0.61 (0.52 - 0.67)
Cluster-wise TPR ↑	0.71 (0.59 - 0.85)	0.80 (0.66 - 0.85)	0.82 (0.74 - 0.87)
Voxel-wise TPR ↑	0.77 (0.70 - 0.89)	0.78 (0.66 - 0.83)	0.78 (0.68 - 0.84)
Voxel-wise FPR ↓	1.8 (1.3 - 5.2) $\times 10^{-4}$	2.0 (1.0 - 4.9) $\times 10^{-4}$	1.9 (1.1 - 4.5) $\times 10^{-4}$

7 Determining domain accuracy at the layer before the lesion label predictor for domain adversarial neural network (DANN) and domain unlearning (DU) models

For DANN and DU models, as an additional test to determine the domain invariance of features at the layer before the lesion label predictor, we performed an experiment to compare the domain accuracy of the models for DANN and DU at this layer. For this experiment, we trained the domain predictor network (shown in figure. S1 with 2 domain labels instead of 5) on the features predicted from DANN and DU (at the layer before the lesion label predictor) for the source and target training data and predicted the domains on the test datasets. During the training phase, we obtained domain accuracies of 54.5% and 53.2% for DU and DANN models respectively on the validation dataset. On the test dataset, we achieved domain accuracies of 58.1% and 57.4% for DU and DANN models respectively. The accuracy value closer to 50% indicates the domain predictor network is unable to differentiate between the two domain since there is not sufficient domain-specific information in the input features. Hence, the results show that, for both the models, the features at the layer before the lesion label predictor are not able to strongly discriminate between domains.

8 Determining the difference between the performance of OXVASC and NDGEN datasets

We considered the evaluation metrics separately for NDGEN and OXVASC datasets and compared them to check for any significance difference between their performance metric values

(using Wilcoxon signed rank test). There was no significance difference between the performance metrics for NDGEN and OXVASC datasets. The performance metrics are tabulated for NDGEN and OXVASC datasets are tabulated in table S5.

Table S5: Comparison of performance metrics for 5 DA test strategies against the target-trained case on NDGEN and OXVASC datasets separately (median and IQR values provided). None of the values are significantly different between the datasets for any strategy.

**Perf. metrics**	**Datasets**	**Strategy 1**	**Strategy 2**	**Strategy 3**	**Strategy 4**	**Strategy 5**	**Target-trained**
SI value ↑	NDGEN	0.80 (0.71 - 0.85)	0.89 (0.62 - 0.96)	0.80 (0.70 - 0.94)	0.89 (0.76 - 0.96)	0.77 (0.70 - 0.83)	0.91 (0.88 - 0.99)
	OXVASC	0.80 (0.70 - 0.84)	0.89 (0.60 - 0.97)	0.78 (0.67 - 0.89)	0.83 (0.77 - 0.95)	0.77 (0.70 - 0.83)	0.86 (0.84 - 0.97)
lAVD ↓	NDGEN	0.20 (0.10 - 0.31)	0.18 (0.04 - 0.27)	0.13 (0.06 - 0.25)	0.08 (0.02 - 0.10)	0.11 (0.03 - 0.14)	0.10 (0.06 - 0.18)
	OXVASC	0.25 (0.10 - 0.33)	0.18 (0.04 - 0.27)	0.12 (0.07 - 0.23)	0.10 (0.04 - 0.13)	0.14 (0.01 - 0.16)	0.09 (0.05 - 0.20)
Cluster-wise F1-measure ↑	NDGEN	0.60 (0.56 - 0.68)	0.62 (0.58 - 0.67)	0.66 (0.61 - 0.85)	0.80 (0.75 - 0.83)	0.69 (0.58 - 0.75)	0.85 (0.81 - 0.99)
	OXVASC	0.56 (0.51 - 0.66)	0.62 (0.57 - 0.65)	0.63 (0.59 - 0.83)	0.78 (0.73 - 0.83)	0.69 (0.58 - 0.75)	0.80 (0.79 - 0.95)
Cluster-wise TPR ↑	NDGEN	0.73 (0.71 - 0.92)	0.83 (0.72 - 0.87)	0.88 (0.86 - 0.99)	0.83 (0.80 - 0.93)	0.78 (0.76 - 0.99)	0.90 (0.88 - 0.99)
	OXVASC	0.70 (0.69 - 0.93)	0.83 (0.72 - 0.87)	0.84 (0.81 - 0.95)	0.80 (0.77 - 0.91)	0.73 (0.70 - 0.94)	0.87 (0.85 - 0.97)
Voxel-wise TPR ↑	NDGEN	0.79 (0.75 - 0.90)	0.83 (0.71 - 0.87)	0.87 (0.82 - 0.95)	0.85 (0.78 - 0.88)	0.82 (0.68 - 0.91)	0.97 (0.94 - 0.99)
	OXVASC	0.76 (0.73 - 0.89)	0.84 (0.71 - 0.87)	0.86 (0.81 - 0.93)	0.88 (0.78 - 0.91)	0.86 (0.63 - 0.99)	0.93 (0.91 - 0.97)
Voxel-wise FPR ↓	NDGEN	2.1 (1.5 - 4.1) $\times 10^{-4}$	1.6 (0.9 - 3.4) $\times 10^{-4}$	1.0 (0.5 - 1.3) $\times 10^{-4}$	1.0 (0.5 - 1.3) $\times 10^{-4}$	1.0 (0.8 - 1.4) $\times 10^{-4}$	1.2 (0.9 - 2.1) $\times 10^{-4}$
	OXVASC	1.5 (1.0 - 4.5)	1.6 (0.9 - 3.3)	0.9 (0.4 - 1.9)	1.2 (0.7 - 1.9)	1.1 (1.0 - 1.8)	1.0 (0.8 - 2.3)

References

[1] K. Simonyan, A. Zisserman. "Very deep convolutional networks for large-scale image recognition." *arXiv* preprint, [online] DOI:arXiv:1409.1556, Sep. 2014.

[2] L. Maaten, and G. Hinton. "Visualizing data using t-SNE." *Journal of machine learning research* vol. 9, pp. *2579 - 2605*, Nov. (2008)

[3] AM. Winkler, GR. Ridgway, MA. Webster, MM. Smith, and TE. Nichols. "Permutation inference for the general linear model." Neuroimage 92 (2014): 381-397.
